# Evidence supports the use of hydrocortisone for patients with community-acquired pneumonia

**DOI:** 10.1186/s13054-024-04833-2

**Published:** 2024-02-20

**Authors:** De-En Lu, Cheng-Yi Chang, Sheng-Wei Cheng, Enoch Kang, Chih-Hsin Lee, Kee-Hsin Chen

**Affiliations:** 1 Division of Nephrology, Department of Internal Medicine, Wan Fang Hospital, Taipei Medical University, Taipei, Taiwan; 2 Division of General Medicine, Department of Internal Medicine, Wan Fang Hospital, Taipei Medical University, Taipei, Taiwan; 3 Division of Gastroenterology, Department of Internal Medicine, Wan Fang Hospital, Taipei Medical University, Taipei, Taiwan; 4 Cochrane Taiwan, Taipei Medical University, Taipei, Taiwan; 5 Evidence-Based Medicine Center, Wan Fang Hospital, Medical University Hospital, Taipei, Taiwan; 6 Institute of Health Policy and Management, College of Public Health, National Taiwan University, Taipei, Taiwan; 7 Pulmonary Research Center, Wan Fang Hospital, Taipei Medical University, Taipei, Taiwan; 8 Division of Pulmonary Medicine, Department of Internal Medicine, Wan Fang Hospital, Taipei Medical University, Taipei, Taiwan; 9 Post-Baccalaureate Program in Nursing, College of Nursing, Taipei Medical University, 252 Wuxing Street, Sinyi District, Taipei, 11031 Taiwan; 10 Department of Nursing, Wan Fang Hospital, Taipei Medical University, Taipei, Taiwan; 11 Research Center in Nursing Clinical Practice, Wan Fang Hospital, Taipei Medical University, Taipei, Taiwan; 12 Evidence-Based Knowledge Translation Center, Wan Fang Hospital, Taipei Medical University, Taipei, Taiwan; 13 School of Medicine, Faculty of Health and Medical Sciences, Taylor’s University, 47500 Subang Jaya, Selangor Malaysia; 14 Division of Pulmonary Medicine, Department of Internal Medicine, School of Medicine, College of Medicine, Taipei Medical University, Taipei, Taiwan

**Keywords:** Community-acquired pneumonia, CAP, Steroids, Corticosteroids, Intensive care unit

Dear editor,

Chiang et al. have given a closer look at the evidence regarding the effects of corticosteroids on severe community-acquired pneumonia (CAP) [1], and the article is a correspondence of a synthesis by Wu et al. [2]. In contrast with the initial synthesis, Dr. Chiang et al. added three relevant trials and distinctively analyzed five studies utilizing hydrocortisone from another set of five studies employing non-hydrocortisone corticosteroids. This stratification was implemented to address concerns related to clinical heterogeneity, despite the fact that the original synthesis already demonstrated minimal statistical heterogeneity in the combined risk ratio for mortality. The original synthesis validated the outcomes through a robust approach involving trial sequential analysis, a step not undertaken in the corresponding analysis. The certainty of the evidence regarding the impact of hydrocortisone on the risk of mortality among patients with CAP remains ambiguous, especially in the absence of sequential analysis, subsequent to the isolation of five studies from the others. The uncertainty could be examined through sequential analysis, reflecting the methodology utilized in the original synthesis—a crucial approach proficient in controlling both Type I and Type II error rates in analyses, ensuring statistical robustness before reaching the required information size [3]. Therefore, this correspondence would like to provide evidence to fulfill the information gap by sequential analysis.

The data presented in the present correspondence were extracted from both the original synthesis and a preceding communication [1, 2]. Our analysis employed methodologies consistent with the previous analyses. Specifically, we utilized the risk ratio (RR) within a random-effects model, applying the DerSimonian–Laird method. The results were reported with a 95% confidence interval (CI). As for the supplementary sequential analysis, it was conducted with a specified type I error of 0.05, a power of 0.80, and a 20% relative risk reduction (RRR), aligning with parameters established in the original synthesis [2]. Incidence in control arm was also obtained from pooled analysis. Meta-analysis procedures were executed using R with the ‘meta’ package version 6.5-0, while sequential analysis was carried out through TSA software (version 0.9.5.10) designed for Microsoft Windows.

Ten studies, encompassing a total of 904 patients in the steroids group and 884 patients in the control group, yielded pooled results indicating a significant reduction in the risk of mortality with steroids compared to control (RR: 0.61; 95% CI 0.43–0.87; Additional file 1: Fig. S1). The acquired information size (AIS) of 1788 exceeded the optimal information size (OIS) of 895, and the cumulative Z-score also surpassed the monitoring boundary (Fig. 1A). However, a noteworthy observation emerged within the subgroup analysis. Specifically, only the hydrocortisone subgroup demonstrated a significant reduction in the risk of mortality (RR: 0.44; 95% CI 0.29–0.65), while a non-significant finding was noted in the non-hydrocortisone subgroup (Additional file 1: Fig. S1). Sequential analysis results further illuminated these findings. In the hydrocortisone subgroup, the cumulative Z-score exceeded the alpha-spending boundary, even though the AIS of 885 did not surpass the OIS of 2155 (Fig. 1B). Conversely, the cumulative Z-score in the non-hydrocortisone subgroup did not reach monitoring boundaries, given a lower AIS of 903 compared to the OIS of 4699. Nevertheless, the result indicated that the cumulative Z-score did not fall into the futility zone (Fig. 1C).

**Fig. 1:**
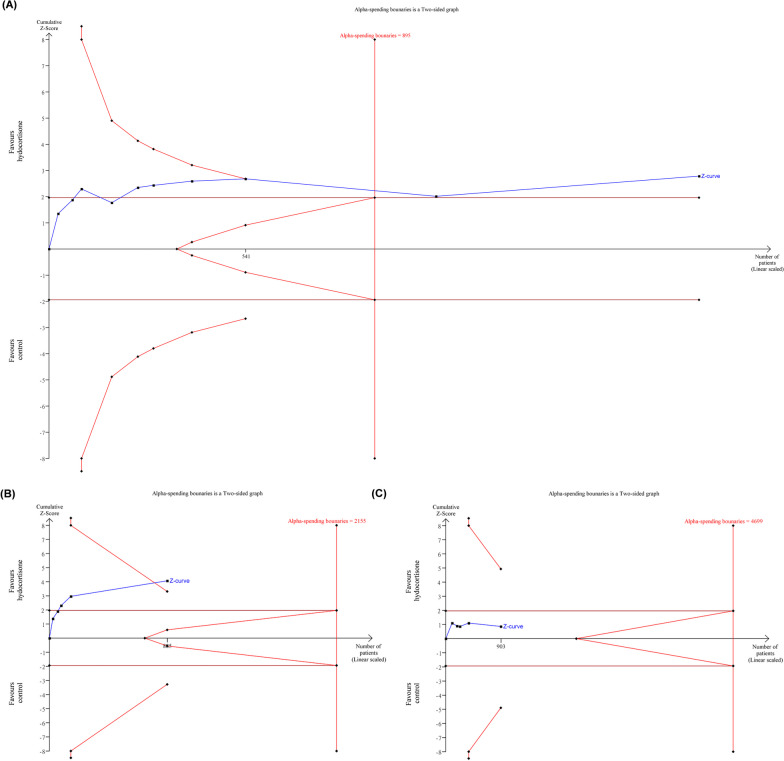
Sequential analysis of mortality using **A** all studies, **B** studies of hydrocortisone, and **C** studies with non-hydrocortisone

This supplementary analysis not only corroborates the findings in both the original synthesis and the preceding communication but also bolsters the confidence in utilizing hydrocortisone for the management of severe CAP [1, 2]. It is important to note, however, that our evidence does not imply ineffectiveness of steroids other than hydrocortisone for patients with severe CAP. The result of sequential analysis for the non-hydrocortisone subgroup should be interpreted cautiously, as the current evidence remains inconclusive. Until clinicians and researchers gain a more comprehensive understanding and evidence regarding the efficacy of steroids other than hydrocortisone in treating severe CAP, it is prudent to consider hydrocortisone as a viable recommendation for the management of patients with CAP. Comparisons between different steroids are worthy of further investigations in the future.

### Supplementary Information

**Additional file 1: Figure S1.** Forest plot of mortality.

## Data Availability

The datasets analyzed in this study are available from the corresponding author on reasonable request.
